# Functional outcome of operative treatment for pelvic metastatic bone disease from primary thyroid cancer: A case series

**DOI:** 10.1016/j.ijscr.2019.11.065

**Published:** 2019-12-10

**Authors:** Yogi Prabowo, Elfikri Asril, Rio Wikanjaya

**Affiliations:** a Consultant of Orthopaedic Oncology, Faculty of Medicine, Universitas Indonesia, Cipto Mangunkusumo Hospital, Jakarta, Indonesia; b Resident of Orthopaedic Surgery, Faculty of Medicine, Universitas Indonesia, Cipto Mangunkusumo Hospital, Jakarta, Indonesia

**Keywords:** Metastatic bone disease, Pelvic, Thyroid cancer, SF-36

## Abstract

**Background:**

Metastatic bone disease (MBD) is the most common malignancy of bone. It is estimated that 70 % of all malignant bone tumors are due to metastasis. The functional outcome from pelvic MBD surgical management has been rarely described in the literature contrary to that of the long bones.

**Method:**

We reported three patients that underwent pelvic resection surgery due to pelvic MBD. All of the primary tumors were thyroid cancer. We measure the functional outcome using SF-36 and MSTS (musculoskeletal tumor society) scores after a one-year follow-up.

**Result:**

The SF-36 scoring result was that all of the patients had a remarkable improvement in terms of pain (77.5 %, 100 %, and 100 %). All of the patients also still had proper social functioning (75 %, 100 %, and 100 %) and good emotional well-being (72 %, 92 %, and 88 %). According to the MSTS, the second and third case had an excellent functional outcome; however the first case had poor functional outcome

**Conclusion:**

Surgery remains the choice of therapy for pelvic MBD that results in alleviation of pain and quality of life improvement.

## Highlights

• 70 % of all malignant bone tumors are metastatic in origin.
• Functional outcome of MBD surgical management rarely described in literature.
• SF-36 and MSTS can be used to measure the functional outcome of MBD surgery.
• Surgery remains a good choice of therapy for MBD of pelvic.

## 1 Introduction

Metastatic bone disease (MBD) is the most common malignancy of bone; it is estimated that 70 % of all malignant bone tumors are metastatic in origin [1]. Primary cancer can spread through the blood or lymphatic circulation to distant organs and metastasize. In theory, any organ in the body may be affected. The bone is the third most common site for metastases, following the lung and the liver. Prostate (32 %), breast (22 %), kidney (16 %), lung, and thyroid cancer have a high risk for MBD. In fact, these primary carcinomas account for 80 % of all the metastases to the bone [2]. There are several biological factors that endorse the bone as a common site of metastasis, such as high blood flow to the red bone marrow, adhesive factors in the bone marrow stroma and the particular environment within the hematopoietic stem cell niche that allows tumor cells to take up residency and dormancy, thereby evading possible effects of systemic therapy [3].

The pelvis is the second most common region of the metastatic bone lesion following the spine. Metastatic tumors of the pelvis may cause pain and a significant loss of function and weight-bearing capacity. Due to the relatively large size of the pelvic cavity, the elastic nature of the organs it contains, and its surrounding muscles, tumors at that site usually reach considerable size before causing symptoms. While some locations of metastases within the pelvis have no impact on pelvic stability and function (e.g., ilium, pubis), tumors of the posterior ilium may pose a threat to lumbosacral integrity, and tumors of the acetabulum may profoundly impair hip function and the weight-bearing capacity of the lower extremity [4]. The MBD of the pelvic is a growing concern in orthopaedic surgery nowadays. Treatment of the pelvic MBD requires a multidisciplinary approach between orthopaedic surgeon, oncologist and radiotherapist [5]. Patients with a pelvic metastasis are individually different with particular needs for management in order to attain the best quality of life despite the advanced stage of disease.^2^ Bone metastases represent the most frequent cause of cancer related pain, affecting health-related quality of life and creating a substantial burden on the healthcare system. Although MBD of the pelvic bone is usually managed conservatively, surgical management can assist in reducing severe pain, restoring structural stability, and reducing the disease burden [6].

The functional outcome affecting health-related quality of life from surgical management of MBD of the pelvic bone has been rarely described in the literature contrary to that of the long bones [6]. In this case series, we reported three cases of pelvic MBD that underwent pelvic resection surgery to investigate the outcomes of surgery as an option for pelvic MBD therapy. This case series has been written according to the PROCESS guideline [7].

## 2 Methods

We reported three patients that underwent pelvic resection surgery due to pelvic MBD. The primary tumors were thyroid cancer. All patients were followed up a minimum of 1 year, and we measured their quality of life by Short-Form 36 (SF-36) and Musculoskeletal Tumor Society (MSTS) score [8,9]. The study was conducted in a national referral hospital. The information was obtained through patient follow-up and medical records. The diagnosis of pelvic MBD was established by careful history taking, physical examination, radiologic examination such as plain radiograph, computed tomography (CT Scan), magnetic resonance imaging (MRI), and either CT guided biopsy or open biopsy for histopathological confirmation. All patients were brought up and discussed in the clinicopathological conference (CPC). The clinical, pathological, and treatment outcomes were obtained according to the SF-36 and MSTS.

### 2.1 SF-36 and MSTS assessment

We obtained the information of all patients from medical records. All patients were called for follow-up, utilizing the SF-36 and MSTS questionnaire. The SF-36 consists of 36 items, grouped into eight scales, each measuring a different aspect of health: physical functioning, role physical (the impact of physical health on performance of everyday role); bodily pain; general health; vitality; social functioning; role emotional (the impact of emotional health on role performance); and mental health. Self-reported health measures provide information about a broader spectrum of health outcomes, and the level of disability associated with them, than the more traditional objective measures of health status such as mortality rates and hospitalization records [8]. Fourteen survey participants completed the SF-36 and MSTS by phone. We called each participant to measure the scoring of SF-36 and the MSTS score.

Case 1 A 45-year-old female with a history of thyroid disease routine consumption of daily levothyroxine for the last ten years complained about right hip pain since three years ago. The pain intensity increased along the time that made her unable to walk. The complaint was accompanied by a lump on her right hip.

**Fig. 1:**
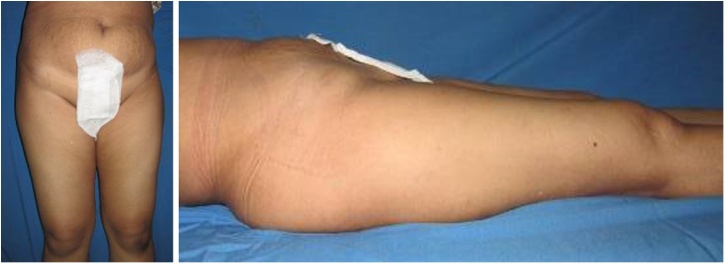
Physical Examination of the Right Hip.

Physical examination of the right hip revealed a lump with a diameter of 12 cm, ill-defined margin, fixed, solid in consistency, slight tenderness (VAS 3–4), the skin temperature was warmer than the surroundings, and limited range of motion of the right hip (Fig. 1).

**Fig. 2:**
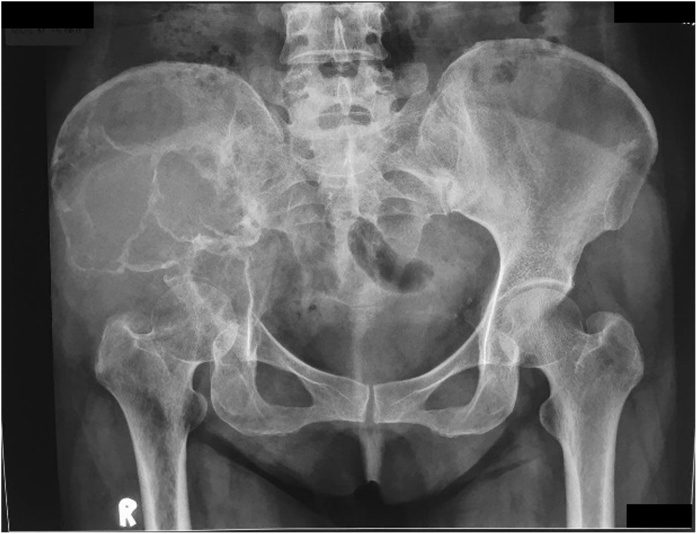
Pelvic x-ray showing destructive lytic lesion involving right ilium and ischium.

**Fig. 3:**
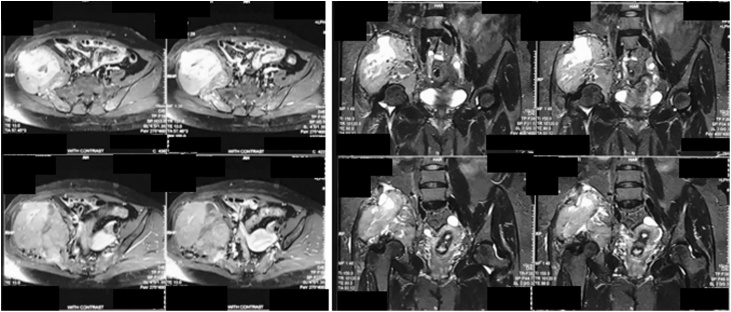
MRI Examination of the Pelvis.

The pelvic x-ray showed a destructive lytic lesion involving the right ilium and ischium (Fig. 2). The thorax x-ray was within normal limits. Furthermore, the MRI result showed an expansile lesion with heterogenic intensity, with an enhancement of the contrast at the right ilium and ischium with muscle involvement. There was no involvement of the neurovascular component (Fig. 3). The patient also underwent bone scan, and a pelvic bone lesion was observed. The laboratory result showed no increase of the alkaline phosphatase (ALP) (83 U/L) and lactate dehydrogenase (LDH) (319 U/L). The free T3 (FT3) was increased (4.86 pg/mL), but the other thyroid markers were normal. Ultrasonography (USG) of thyroid was also performed and revealed a multinodular goiter with cystic degeneration, with hypervascularization was also observed. A CT-guided biopsy was conducted on the lump on right hip and revealed bone metastasis from thyroid cancer. The case was brought up in the CPC, and the diagnosis was established. Neck biopsy revealed papillary carcinoma thyroid.

**Fig. 4:**
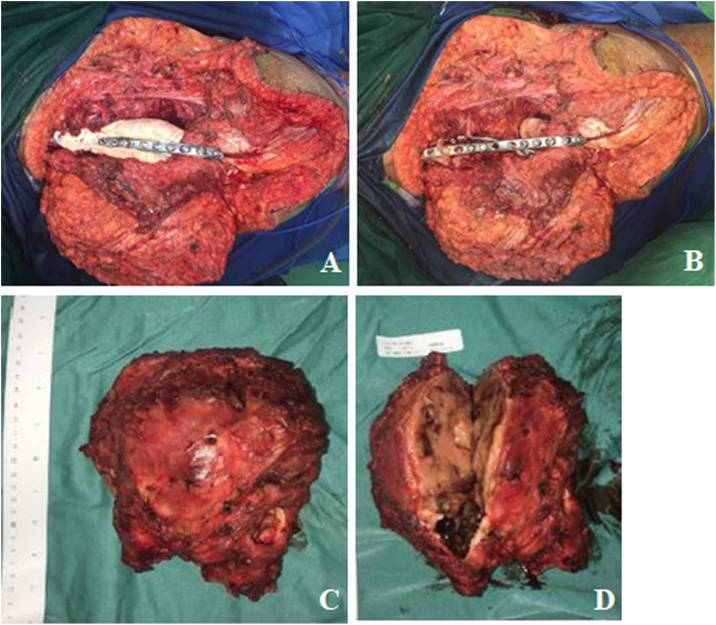
Intraoperative Procedure. **(**A)(B) reconstruction of the periacetabular region with plate, screw, and bone cement, (C)(D) gross pathology.

**Fig. 5:**
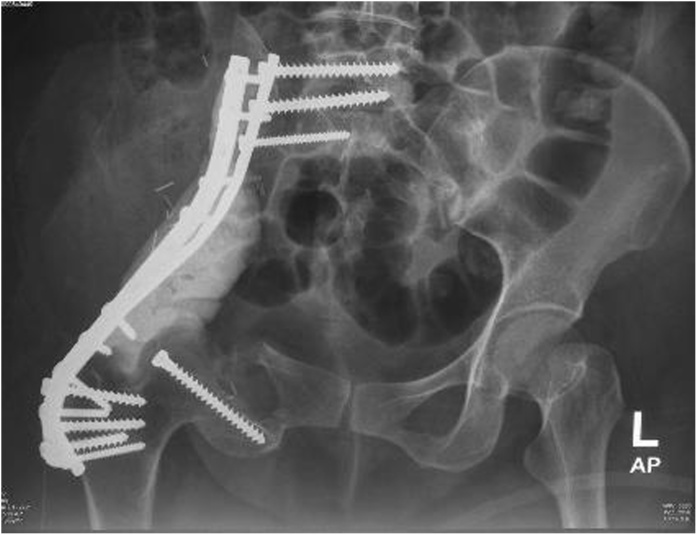
Postoperative X-Ray Examination Showing Plate, Screw, and Bone Cement after Reconstruction Procedure.

We decided to perform type I and II pelvic resection and hip arthrodesis for this patient. Preoperatively, we did an embolization to decrease the risk of bleeding. We excised the tumor along with the periacetabular region intraoperatively and reconstructed it with a plate, screws, and bone cement (Fig. 4). Postoperatively the patient was observed in ICU for one day. She had excellent hemodynamics and was discharged five days after surgery (Fig. 5).

**Fig. 6:**
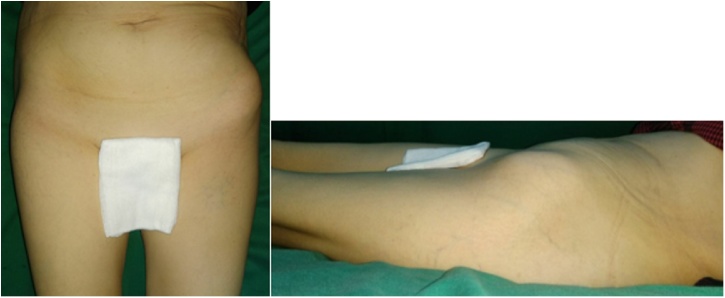
Physical Examination of the Patient.

Case 2 A 55-year-old female with left hip pain that accompanied by a lump for one year was referred to our institution. The pain intensity increased along the time, but the patient was still able to walk. There were no complaints regarding micturition nor defecation, and there was no decrease in motoric nor sensory functions. Previously she was already diagnosed with a thyroid tumor and consumed PTU three times daily for last three years (Fig. 6).

Physical examination of the pelvic region revealed a lump on the left hip 10 × 9 cm in size, fixed, solid, tenderness VAS 3–4, normal sensory, and limited hip range of motion (ROM) due to pain. LDH and ALP values were within normal limits; the thyroid markers were normal for total T3 (1.8 pg/mL) and decreased total T4 (1.5 mcg/dL).

**Fig. 7:**
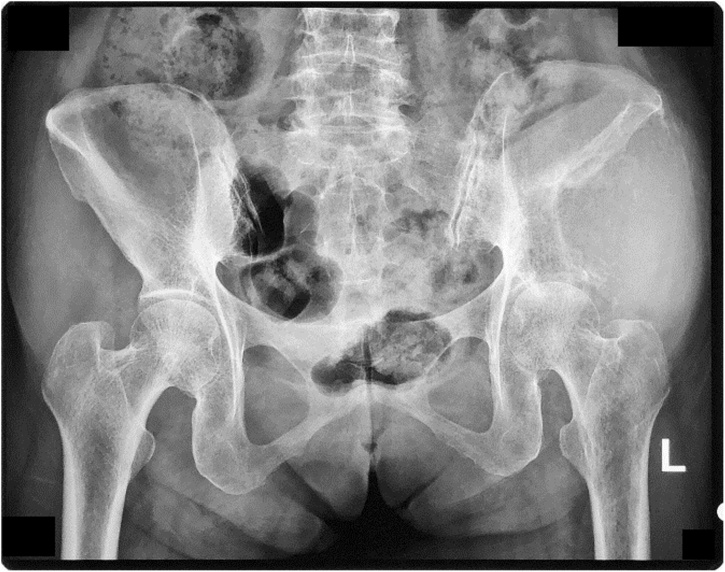
Pelvic Radiography Showing Destructive Mass.

**Fig. 8:**
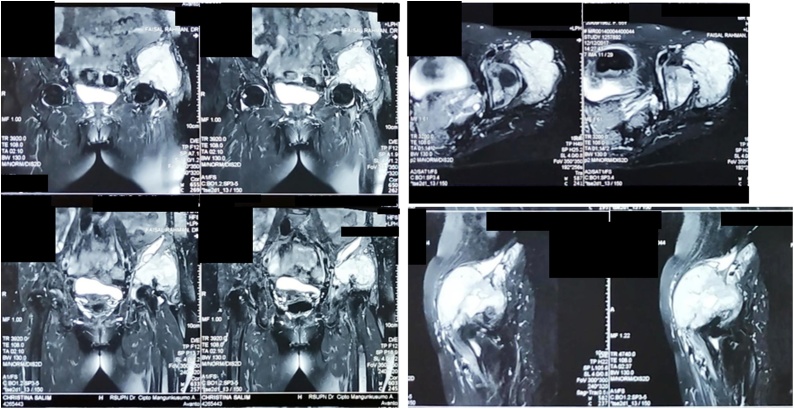
MRI Examination of the Pelvic.

From the pelvic radiography, there was a mass with left iliac bone destruction until acetabulum, that suggested metastasis (Fig. 7). MRI examination of the pelvis showed a solid mass with a necrotic component on the left iliac wing extending to the left acetabulum involving the iliac and gluteus minimus muscle; there was also multiple lesion at the femoral head to the left proximal shaft femur (Fig. 8). The patient underwent core biopsy, and tumor cells arranged in groups with papillar structure suitable with metastasis lesion was observed under the microscope. A core biopsy of the neck tumor revealed papillary thyroid carcinoma (Table 1).

**Table 1:** Demographic Data of the Included Patients.

No	Sex	Age	Primary Tumor	treatment	Functional Outcome	
					SF 36	MSTS
1	F	44	Thyroid Cancer	Pelvic Resection type I and II + hip arthrodesis	Physical functioning: 0 %	8
					Role limitations due to physical health: 0 %	
					Role limitations due to emotional problems: 0 %	
					Energy/fatigue: 70 %	
					Emotional well-being: 72 %	
					Social functioning: 75 %	
					Pain: 77.5 %	
					General health: 40 %	
					Health change: 75 %	
2	F	55	Thyroid Cancer	Pelvic Resection type I and II + THR	Physical functioning: 85 %	27
					Role limitations due to physical health: 75 %	
					Role limitations due to emotional problems: 100 %	
					Energy/fatigue: 85 %	
					Emotional well-being: 92 %	
					Social functioning: 100 %	
					Pain: 100 %	
					General health: 75 %	
					Health change: 100 %	
3	F	37	Thyroid Cancer	Pelvic resection type II and THR	Physical functioning: 90 %	30
					Role limitations due to physical health: 100 %	
					Role limitations due to emotional problems: 100 %	
					Energy/fatigue: 90 %	
					Emotional well-being: 88 %	
					Social functioning: 100 %	
					Pain: 100 %	
					General health: 50 %	
					Health change: 100 %	

**Fig. 9:**
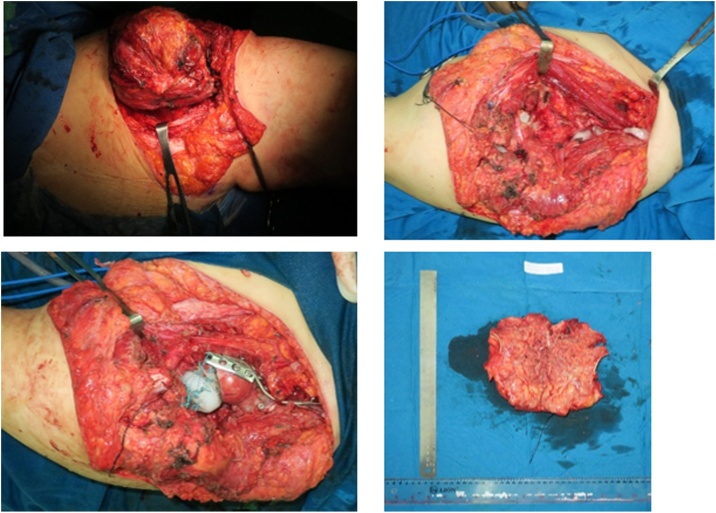
Intraoperative Procedure of the Patient.

**Fig. 10:**
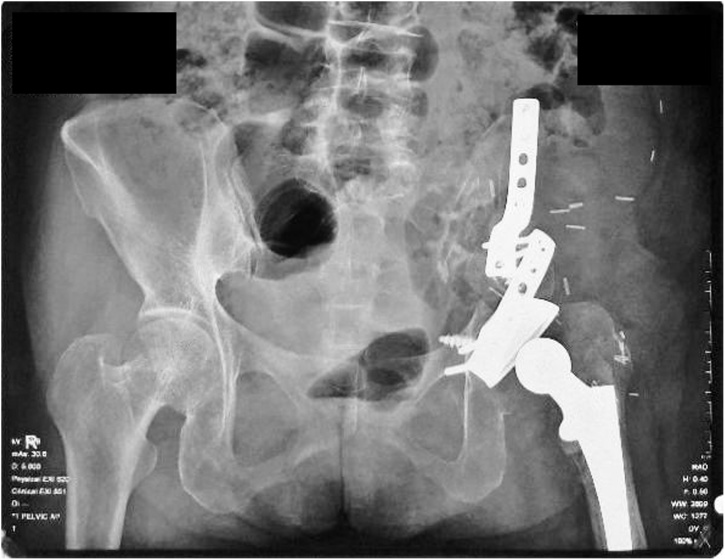
Postoperative Pelvic X-Ray Examination.

After being discussed in the clinicopathological conference, this patient was diagnosed with pelvic MBD from thyroid cancer. We exposed and excised the tumor along with the periacetabular region; reconstruct using THR, plate, screws, and bone cement (Fig. 9). Acetabuloplasty using femoral head was conducted. Later, the patient was observed in ICU for one day; the hemodynamics was within normal limited, and she was discharged five days after surgery (Fig. 10).

**Fig. 11:**
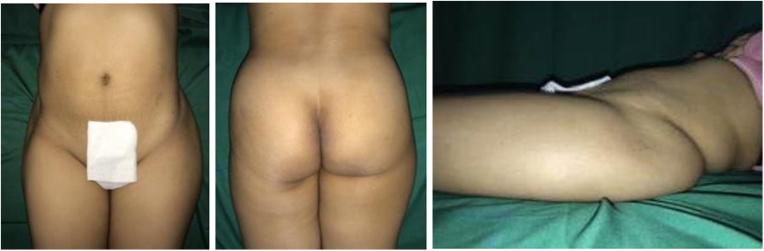
Physical Examination of the Patient.

Case 3 A 37-year-old female previously diagnosed with a thyroid tumor for three years complained of left hip pain since one year ago. The pain got worsen progressively especially during the night. She is still able to walk however with an antalgic gait. Physical examination of the left hip found no mass nor deformity. However, a tenderness that limits her range of motion was present. Laboratory examination showed normal LDH (310 U/L) and ALP (66 U/L). The FT4 was decreased (0.590 ng/dL) while other thyroid markers were normal (Fig. 11).

**Fig. 12:**
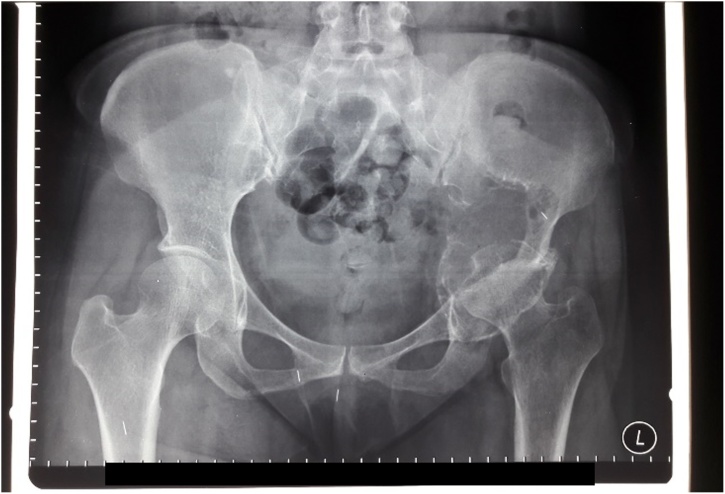
Pelvic X-Ray Examination Showing Lytic Lesion on the Left Ilium and Ischium.

**Fig. 13:**
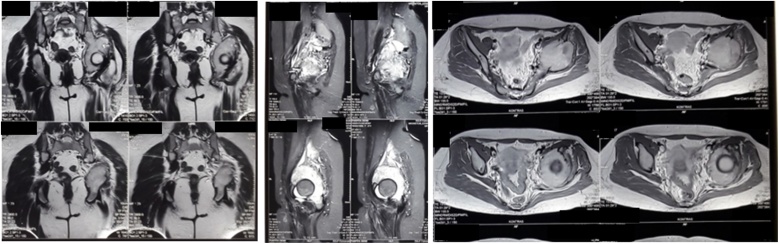
MRI Examination of the Pelvis.

Pelvic AP radiograph revealed a lytic lesion on the left ilium and ischium, involving the left acetabulum that suggested a malignant tumor (Fig. 12). Contrast MRI of the pelvis showed a solid mass on the ilium, ischium, and the left acetabulum, with solid density and minimal cystic component (Fig. 13).

**Fig. 14:**
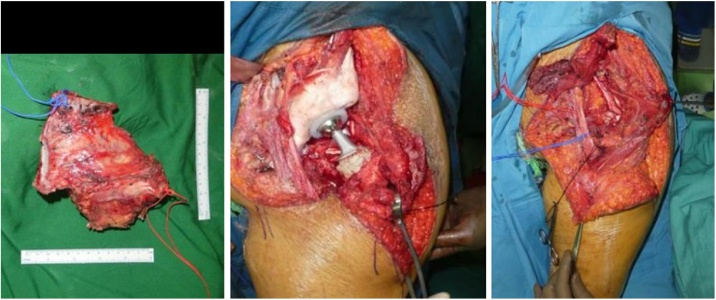
Intraoperative Procedure of the Patient.

**Fig. 15:**
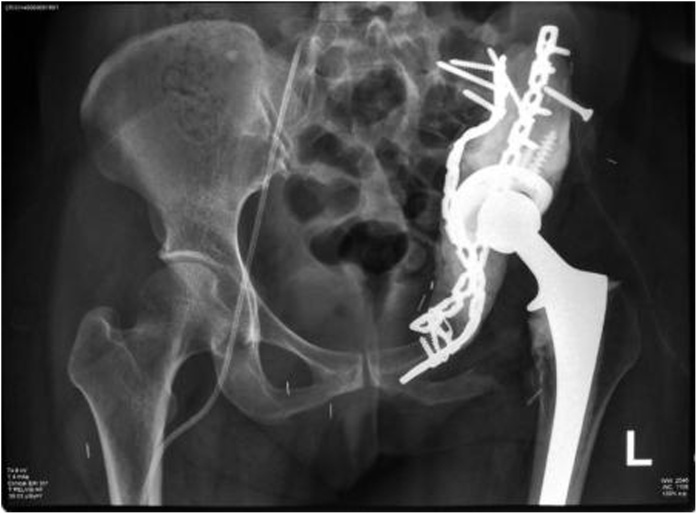
Postoperative X-Ray Imaging Showing Plate, Screw, and Total Hip Replacement.

A core biopsy was performed, and the results showed thyroid cancer metastasis. Biopsy from the thyroid gland showed papillary carcinoma type. The case was brought up in the CPC and based, the patient was diagnosed with pelvic MBD. We performed type II pelvic resection and total thyroidectomy (performed by a head, neck, and breast surgery consultant). We performed an osteotomy and reconstruction using plate, screws, along with THR (Fig. 14). Postoperatively the patient was observed in ICU for one day. The hemodynamics was within normal limits, and she was discharged five days after surgery (Fig. 15).

## 3 Result

**Fig. 16:**
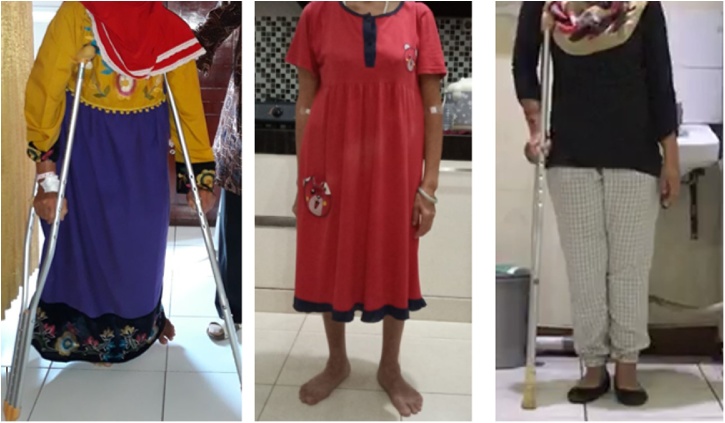
Patient’s clinical picture after a one-year follow-up. A. 1 st case, B. 2nd case, C. 3rd Case.

All of our patients were middle-aged female patients aged 37–55 years old. All patients had primary thyroid cancer and underwent pelvic MBD surgery in concordance with the respecting types. MBD of the first and second case was located in periacetabular to the iliac wing so that we did pelvic resection type I and II. Meanwhile, we performed type II resection in the third case as the tumor was located in the periacetabular region only. For the reconstruction after pelvic resection, we performed THR and plate-and-screw fixation for second and third cases; and hip arthrodesis and plate screw for first case. All operative procedures were performed by oncology and reconstruction consultants with experience more than ten years (Fig. 16).

The patients were followed up for a minimum of one year; we measured functional outcomes of patients using SF-36 and MSTS scores. The SF-36 score result was that all of the patients had good improvement in terms of pain (77.5 %, 100 %, and 100 %, respectively). All of the patients also still had proper social functioning (75 %, 100 %, and 100 %, respectively) and good emotional well-being (72 %, 92 %, and 88 %, respectively). There was a limitation of physical function on the first patient, but practically none in the second and third cases. According to the MSTS scoring, the second and third case was categorized as having a very good functional outcome. One patient could walk independently, and two patients could walk with walker. In general, after surgery the quality of life of the patients was improved. We also evaluated the local recurrence of the tumor, and after a one-year follow-up, we did not find any local recurrence in these patients, which was assessed by performing interviews and physical examinations along with x-ray and MRI during follow up.

## 4 Discussion

In our study, all cases were primary from thyroid cancer, which is in concordance to the study conducted by Krishnan et al. that the most common primary tumor for MBD pelvis is thyroid cancer [6]. All patients had papillary thyroid cancer, which is the most common thyroid malignancy [10]. Papillary tumor is found in approximately 80 % of all thyroid malignancy and occurs mostly in the third to the fifth decades of life [10,11]. Interestingly, papillary thyroid cancer has been reported to have lower tendency to metastasize distally than follicular type [11]. Bone metastasis in follicular thyroid cancer ranges from 7 to 28 % meanwhile the rate for papillary thyroid cancer is 1.4–7 % [12]. Since the number of papillary thyroid cancer patients is higher, some study argues that the number papillary thyroid cancer patient will be higher in term of bone metastasis. The study conducted by Tickoo et al. reported that the number of bone metastases was higher in papillary than follicular thyroid cancer [11]. In this study all of metastases bone cases were located in periacetabular to iliac wing region which is also similar with Krishnan’s study that found the most common site of MBD pelvic were in iliac wing and periacetabular region [6].

The treatment of MBD in the pelvic region requires a multidisciplinary approach between orthopaedic surgeons, oncologists, and radiotherapists. The treatment depends on the patient’s prognosis, the exact site of the metastasis in the pelvis, and the amount of bone loss of the periacetabular region. The goals of surgical treatment of metastases to the pelvic bone are similar to those for MBD at any other site, which includes local tumor control, structural stability, and restoration of function. For the patient who does not benefit from surgical resection of the metastatic lesion, they are conservatively treated to improve quality of life [5].

Although the exact indication of surgery is still debatable, Jehn and colleagues also proposed a non-vertebral MBD treatment algorithm with palliative, preservation, and restoration of function. Non-vertebral MBD should be surgically treated as pathological fractures, even in advanced disease. An impending fracture of a long bone or pelvic bone warrants a prophylactic fixation followed by postoperative radiation therapy. Jehn et al. recommend prophylactic radiation for asymptomatic nonvertebral bone metastasis to increase local tumor control. If multiple symptomatic non-vertebral bone metastases are present, treatment with therapeutic radioisotopes can be considered [13]. Capana and Campanacci also introduced algorithm for pelvic bone metastases by classifying the patients into four groups; those with (1) solitary lesion with good prognosis, (2) pathologic fracture in periacetabular region, (3) supraacetabular osteolytic lesion and (4) multiple osteoblastic lesion at all sites, osteolytic or mixed lesions in iliac wing and anterior pelvis [5]. Group 1 is treated as a primary tumor, and the operation aims to achieve a long term cure, both biological and mechanical. The treatment of group 2 is aimed to restore mechanical integrity and function if the fracture already occurred. The treatment goal for group 3 is to prevent a pathological fracture. Meanwhile, in group 4 should be treated conservatively by chemotherapy, hormonal therapy and irradiation according to the diagnosis [2].

Pelvic resection was performed in all cases in this report. The type of pelvic resection depends on the location of the tumor. The pelvic bone loss due to tumor and resection need to be reconstructed to restore the femorosacral continuity for weight-bearing because the pelvic itself has function to transmit the weight of upper body to the lower extremity and contains the hip joint [14,15]. We reconstructed the pelvic using total hip replacement (THR) for second and third case and hip arthrodesis for the first case along with bone cement, plates, and screws. We performed THR to preserve joint motion. However, we did hip arthrodesis in the first case due to an inadequate amount of remaining ilium and pubis for fixation. We suppose the hip arthrodesis restricts the hip ROM and activities; therefore, is postulated as the primary cause of the lower MSTS score compared to the other two patients.

The functional outcome was measured using MSTS and SF-36. The results showed that there was an improvement in pain, social life, and the psychological condition of the patients. Slight limitation of function was present. This result corresponds to the study conducted by Singh et al. and Krishnan et al. that stated quality of life improvement after surgery in patients with MBD pelvis [6].

## 5 Conclusion

To conclude, surgery remains the right choice of therapy for MBD of the pelvis that resulted in the alleviation of pain and improvement in the quality of life. This study is limited by the number of patients as pelvic MBD is considered uncommon in general. The follow up period of one year might also be considered too short to observe outcomes. A prospective cohort can be recommended to further investigate the long-term outcome of surgery in pelvic MBD.

## Declaration of Competing Interest

The authors declare that there is no conflict of interest regarding the publication of this paper.

## Funding

The authors received no financial support for the research, authorship, and/or publication of this article.

## Ethical approval

The ethical approval was not required for this case series.

## Consent

Written informed consent was obtained from all of the patients for publication of this case report and accompanying images. A copy of the written consent is available for review by the Editor-in-Chief of this journal on request.

## Author contribution

Yogi Prabowo: Concept of the study, data collection & interpretation, and writing the paper.

Elfikri Asril: Data collection, data interpretation and writing the paper.

Rio Wikanjaya: Data collection, data interpretation and writing the paper.

## Registration of research studies

UIN number: researchregistry5109.

## Guarantor

Yogi Prabowo.

## Provenance and peer review

Not commissioned, externally peer-reviewed.
